# STING Contributes to Host Defense Against *Staphylococcus aureus* Pneumonia Through Suppressing Necroptosis

**DOI:** 10.3389/fimmu.2021.636861

**Published:** 2021-05-31

**Authors:** Zhen-Zhen Liu, Yong-Jun Yang, Cheng-Kai Zhou, Shi-Qing Yan, Ke Ma, Yu Gao, Wei Chen

**Affiliations:** Key Laboratory of Zoonosis Research, Ministry of Education, College of Veterinary Medicine, Jilin University, Changchun, China

**Keywords:** *Staphylococcus aureus* (MRSA), pneumonia, STING, innate immune, necroptosis

## Abstract

STING (Stimulator of interferon genes) is known as an important adaptor protein or direct sensor in the detection of nucleotide originating from pathogens or the host. The implication of STING during pulmonary microbial infection remains unknown to date. Herein, we showed that STING protected against pulmonary *S.aureus* infection by suppressing necroptosis. STING deficiency resulted in increased mortality, more bacteria burden in BALF and lungs, severe destruction of lung architecture, and elevated inflammatory cells infiltration and inflammatory cytokines secretion. STING deficiency also had a defect in bacterial clearance, but did not exacerbate pulmonary inflammation during the early stage of infection. Interestingly, TUNEL staining and LDH release assays showed that STING^-/-^ mice had increased cell death than WT mice. We further demonstrated that STING^-/-^ mice had decreased number of macrophages accompanied by increased dead macrophages. Our *in vivo* and *in vitro* findings further demonstrated this cell death as necroptosis. The critical role of necroptosis was detected by the fact that MLKL^-/-^ mice exhibited decreased macrophage death and enhanced host defense to *S.aureus* infection. Importantly, blocking necroptosis activation rescued host defense defect against *S.aureus* pneumonia in STING^-/-^ mice. Hence, these results reveal an important role of STING in suppressing necroptosis activation to facilitate early pathogen control during pulmonary *S.aureus* infection.

## Introduction

*Staphylococcus aureus* (*S.aureus*) is a leading cause of pneumonia and particularly as a secondary infection of influenza (1, 2). The methicillin-resistant *S.aureus* (MRSA) USA300 strain causes a widespread infection of the skin, soft tissues, and lung, these infections are related to high morbidity and mortality (3, 4). Furthermore, *S.aureus* has its arsenal of virulence factors to develop antibiotic resistance. Therefore, *S.aureus* is a serious threat to human health and requires effective treatment strategies.

Innate immunity is the first line of defense against pathogens invasion. Host cells have equipped with a majority of pattern recognition receptors (PRRs), including Toll-like receptors (TLRs), NOD-like receptors (NLRs), C-type lectin receptors, cytosolic DNA sensors, and RIG-I-like receptors, recognizing invading microbes and initiating appropriate immune responses (5). Concerning *S.aureus*, TLR2 (6), TLR8 (7), TLR9 (8), NOD2 (9), and IFI204 (10) were implicated in *S.aureus* recognition. Moreover, growing evidence indicates that *S.aureus* has evolved to utilize host innate immune molecules to evade eradication by the host immune system, as mice deficient in NLRC4, NLRP6, NOD2, TNFR, and IFNAR expression exhibited significantly improved outcomes in *S.aureus* pneumonia (11–15), mainly reflected in reduced bacterial burden and attenuated pro-inflammatory cytokine production which contributing to increased survival. Hence, investigating the innate immune mechanisms of host resistance to *S.aureus* is conducive to the development of novel strategies for control of this infection.

STING also known as TMEM173, ERIS, MITA, or MPYS. This endoplasmic reticulum-associated protein recognizes cyclic dinucleotides (CDNs), more importantly, STING also as an adaptor protein for diverse DNA sensors including IFI204, cGAS, ZBP1, and DDX41. While it is activated, STING serves as a scaffold for TBK1 and the transcription factor IRF3 (16, 17). IRF3 is phosphorylated by TBK1, after which it translocates into the nucleus to stimulate the transcription of type I interferons (18). STING-mediated type I interferons signaling mainly participates in effective immune responses against viral infections. Its role during bacterial diseases is controversial, ranging from protective to detrimental effects for the host (19). Particularly, IFNAR-deficient mice were sufficiently protected against *S.aureus* pneumonia compared with WT mice, consistent with our previous study that administration of recombinant IFN-β promotes *S.aureus* proliferation in IFI204^-/-^ mice (10, 20–22). STING is involved in diverse bacterial infections and exerts different immune responses depending on pathogens and different infectious models. Studies have demonstrated that STING played a protective role in response to bacterial infections such as *Pseudomonas aeruginosa* (23), *Listeria monocytogenes* (24), *Brucella* (25), and *Mycobacterium tuberculosis* (26, 27). Especially, in the model of *S.aureus* cutaneous infection, activation of STING antagonized innate immunity and resulted in infection spread through decreased neutrophil recruitment and IL-1β secretion (28). However, much less is known about the biological implication of STING in host defense specifically during pulmonary microbial infection. After all, the route of administration of bacteria also determines host diverse responses.

In the current study, we show that STING plays a critical role in pulmonary defense against *S.aureus* infection. STING-deficient mice exhibit higher mortality rates, more bacterial colonization, and more severe lung damage compared to control mice. STING promotes early pulmonary clearance of *S.aureus* before developing excessive inflammation. STING deficiency results in increased necroptosis which facilitates bacterial pulmonary proliferation. Blocking necroptosis rescues the defect of pathogen control in STING^-/-^ mice. Collectively, our results suggest that STING is essential for the host defense against pulmonary *S.aureus* infection through inhibiting necroptosis activation.

## Materials and Methods

### Mice

C57BL/6J wild-type (WT) mice and STING^-/-^ mice were purchased from the Jackson Laboratory (Bar Harbor, ME, USA). MLKL^-/-^ mice were a gift from Dr. Jia-Huai Han, Xiamen University, China. They were subsequently backcrossed onto the C57BL/6J background at least eight generations. Age and gender matched WT controls were used. The animal studies were conducted according to the experimental practices and standards approved by the Animal Welfare and Research Ethics Committee at Jilin University (No. 20150601).

### Pneumonia Model

To induce pneumonia, mice were anesthetized with pentobarbital sodium (50 mg/kg, i.p.) prior to intranasal inoculation of *S.aureus* (USA300 strain), twenty μL of bacterial suspension containing 1 × 10^8^ CFU of log-phase *S.aureus* in phosphate-buffered saline (PBS) was inoculated intranasally to each mouse, subsequently rinsed with ten μL of PBS. At 6 h and 24 h post-infection, mice were euthanized to collect bronchoalveolar lavage fluid (BALF) and lung for quantification of bacterial burden. BALF was collected by instilling inside the lung with 0.8 mL PBS containing 100 µg/mL soybean trypsin inhibitor, which process repeated three times. Cell counts were performed on BALF using light microscopy utilizing Quik-Dip Stain. A part of the lung was homogenized mechanically in cold PBS (at a ratio of 4 mL per gram tissue). BALF and homogenized lung were serially diluted and plated onto TSB agar plates for bacterial enumeration. For survival experiments, we used 2 × 10^8^ CFUs/mouse of *S.aureus* and observed survival for 80 h post-infection. In other experiments, 100 μL of 100 μM of GW806742X (MLKL inhibitor) (SYN-1215, Synkinase, San Diego, CA, USA) was administered intraperitoneally 12 h and 1 h prior to infection. Control mice received equal DMSO.

### MPO Assay

Lungs collected from WT and KO mice after *S.aureus* infection were homogenized in 0.5% cetyltrimethylammonium chloride (4 µL/mg lung). The supernatants were obtained for MPO activity assay. Briefly, samples were transferred to a 96-well plate mixing with equal volumes (75 µL) of the substrate (3,3’, 5,5’ tetramethyl-benzidine dihydrochloride, 3 mmol/L; resorcinol, 6 mmol/L; and H_2_O_2_, 2.2 mmol/L) for 2 min. 150 µL of 2 mol/L H_2_SO_4_ was used to stop the reaction. The OD was measured at 450 nm.

### Tissue Histology and Immunostaining

Lung tissues were fixed in 4% paraformaldehyde and embedded in paraffin. For histology, lung sections (5 μm) were stained with hematoxylin and eosin (H&E). For immunohistochemistry, sections were stained with STING (19851-1-AP, Proteintech, Wuhan, Hubei, China) and Ly-6G/Ly-6c (BioLegend, San Diego, CA, USA) antibodies. For immunofluorescence, cells were stained with p-MLKL primary antibody (ab196436, Abcam, Cambridge, MA, USA), and CoraLite594-conjugated secondary antibody (SA00013-4, Proteintech, Wuhan, Hubei, China). Subsequently, pulmonary cell death was analyzed by TUNEL staining using a commercial kit (KGA702, KeyGEN BioTECH, Beijing, China). DAPI (1 µg/mL) was used to stain nuclei.

### Cytokine and Chemokine Measurements

To measure the cytokine and chemokine amounts in lung tissue, a part of the lung was homogenized mechanically in cold PBS (at a ratio of 4 mL per gram tissue) containing 1% Triton X-100 and a complete protease inhibitor cocktail (P8340, Sigma-Aldrich, St. Louis, MO, USA). The ELISA kits were purchased from R&D Systems. Cytokines and chemokines in lung tissue or cell supernatants were measured by ELISA according to the manufacturer’s instructions.

### Real-Time PCR

RNA was isolated using TRI reagent (Sigma-Aldrich) and converted into cDNA. Subsequently, real-time PCR assays were performed using SYBR Green (Roche, Basel, Switzerland) on an ABI Prism 7500 sequence detection system (Life Tech [Applied BioSystems], Waltham, USA). Gene expression levels were calculated using the 2^−ΔCt^ method. The following primer sequences were used: GAPDH sense 5’-CACCCCAGCAAGGACACTGAGCAAG-3’ and antisense 5’-GGGGGTCTGG GATGGAAATTGTGAG-3’. IFN-β sense 5’-ACTGCCTTTGCCATCCAAGA-3’ and antisense 5’-CACTGTCTGCTGGTGGAGTT-3’.

### Flow Cytometry

Cells in BALF were centrifuged at 515 × g for 5 minutes. For flow cytometry analysis, red blood cell lysis buffer was used to removed red blood cells and remaining cells washed with PBA buffer. Cells were suspended in 100 μL PBA Buffer (1% bovine serum albumin and 0.1% sodium azide in PBS) and stained for 1 h at 4°C from light. Combinations of fluorescein allophycocyanin (APC)-labelled anti-F4/80 (123115, Biolegend, San Diego, CA, USA) and propidium iodide (PI, Life Technologies) were used. Dead macrophages (F4/80^+^/PI^+^) were analyzed in a FACSAria flow cytometer (BD Biosciences).

### Western Blotting

BMDMs or lungs were harvested and then homogenized in basic RIPA buffer solution containing 1% Triton X-100, 50 mM Tris-HCl (pH 8.0), 150 mM NaCl, 0.25% Sodium deoxycholate, and 0.1% SDS, which was added complete protease inhibitor cocktail (P8340, Sigma-Aldrich, St. Louis, MO, USA). Total cell lysates were separated by SDS-PAGE and transferred to PVDF membrane. After blocking with 5% milk, the membranes were incubated with primary antibodies against RIPK3 (AP7819b, ABGENT, San Diego, CA, USA), p-MLKL (ab196436, Abcam, Cambridge, MA, USA), MLKL (MABC604, Millipore, Billerica, MA, USA), and GAPDH (10494-1-AP, Proteintech, Wuhan, Hubei, China).

### Cell Culture and Infection

Bone marrow derived macrophages (BMDMs) were sterilely isolated from femurs of 6-8-week-old mice and cultured in RPMI-1640 (Gibco, Waltham, MA, USA) containing 10% FBS (Hyclone, Logan, UT, USA), 25% L929 cell-conditioned medium, 100 U/mL penicillin, and 100 U/mL streptomycin. Cells were harvested for assays at day 7 of differentiation. BMDMs were infected with *S.aureus* at a multiplicity of infection (MOI: 50), centrifuged at 515 × g for 2 min for synchronous infection, incubated at 37°C for the designated time points. The cell supernatants and lysates were collected for ELISA or western blotting assay.

### Measurement of Cell Death

BMDMs were isolated from WT and STING^-/-^ mice and pretreated with Nec-1 (50 μM) or DMSO for 1 h before infection with *S.aureus* (MOI: 50). The percentage of cytotoxicity in BMDMs was measured by PI staining at the designed time points. The dead cells are stained red. LDH release into the alveolar space after *S.aureus* infection was evaluated using the CytoTox 96 Non-Radioactive Cytotoxicity Assay Kit (G1782, Promega, Madison, WI, USA).

### Statistical Analysis

Data are represented as the mean ± SEM. Data sets with only two independent groups were analyzed for statistical significance using unpaired, two-tailed Student’s t-test. Data sets with more than two groups were analyzed using one-way ANOVA with Bonferroni’s multiple comparison test. All P values less than 0.05 were considered significant (* P < 0.05, ** P < 0.01, *** P < 0.001). Statistical analysis was performed using Prism (GraphPad Software, La Jolla, CA, USA).

## Results

### STING Contributes to Host Protection Against *S.aureus* Pneumonia

**Figure 1:**
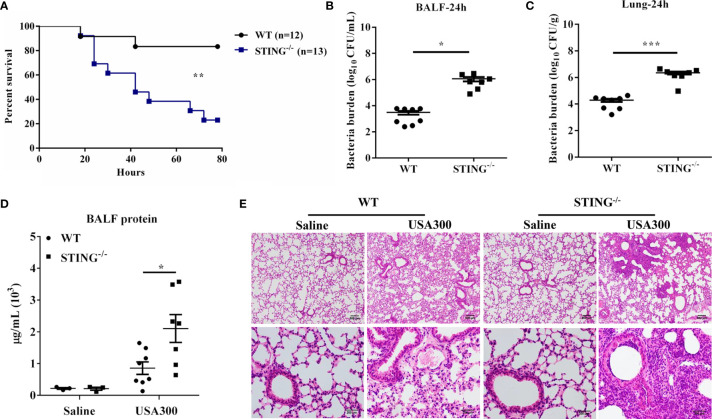
STING-deficient mice display increased susceptibility to *S.aureus* pulmonary infection. WT and STING^-/-^ mice were infected intranasally with *S.aureus*. **(A)** Survival was monitored up to 80 hpi (2 × 10^8^ CFU, N = 12-13/group). WT and STING^-/-^ mice (N = 7-8/group) were infected intranasally with a sublethal inoculum of *S.aureus* (1 × 10^8^ CFU/mouse) for 24 h and then euthanized to quantitate the bacterial burden in **(B)** BALF, **(C)** lung. **(D)** Total protein in BALF was measured. **(E)** Lung tissue structures were observed by hematoxylin and eosin staining (magnification of 100 × or 400 ×). All data are shown as mean ± SEM. Student’s t-test was performed. Log-rank test was used for statistical analysis of animal mortality. Statistical significance is indicated by *p < 0.05, **p < 0.01, and ***p < 0.001.

To assess the role of STING in pulmonary host defense against *S.aureus* infection, WT and STING^-/-^ mice were intranasally challenged with a lethal dose of *S.aureus* (2 × 10^8^ CFUs per mouse). The animals were monitored for 80 h after infection. Compared to WT counterparts, STING^-/-^ mice showed decreased survival (**Figure 1A**). To determine if the decreased survival in STING^-/-^ mice was due to increased bacterial burden, WT and STING^-/-^ mice were infected intranasally with a sub-lethal inoculum of *S.aureus* (1 × 10^8^ CFUs per mouse). STING^-/-^ mice had increased bacterial burden in the lung and bronchoalveolar lavage fluid (BALF) compared to WT mice at 24 h post infection (hpi) (**Figures 1B, C**). Barrier function, as measured by protein content in BALF, was obviously disrupted (**Figure 1D**), and lung architecture was more severe in the STING^-/-^ mice (**Figure 1E**). Collectively, these results suggested that STING plays an indispensable role for protection against *S.aureus* pneumonia.

### STING Deficiency Results in Increased Inflammatory Responses Following Pulmonary *S.aureus* Infection

**Figure 2:**
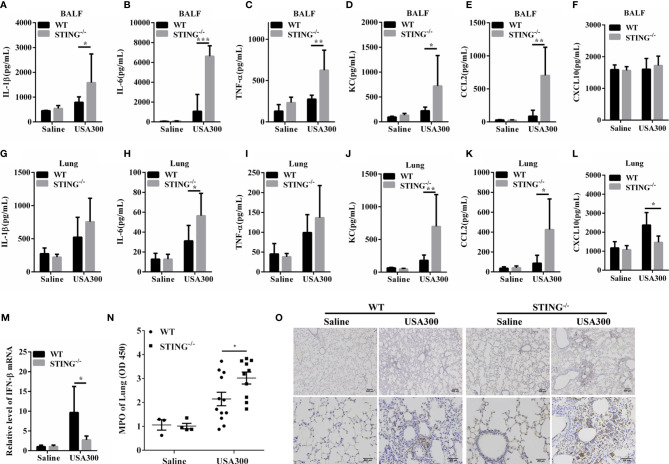
STING deficiency leads to enhanced pro-inflammatory mediator production following *S.aureus* lung infection. WT and STING^-/-^ mice (N = 7-8/group) were infected intranasally with *S.aureus* (1 × 10^8^ CFU/mouse) for 24 h. The homogenate supernatants of lungs and BALF were detected for concentrations of indicated cytokines and chemokines by ELISA. **(A, G)** IL-1β, **(B, H)** IL-6, **(C, I)** TNF-α, **(D, J)** KC, **(E, K)** CCL2, **(F, L)** CXCL10. **(M)** Lung tissue mRNA was examined for IFN-β by qRT-PCR. **(N)** The homogenate supernatants of lungs were also used to determine the activity of MPO (a neutrophil marker). **(O)** Representative immunohistochemical staining of Gr-1 (a neutrophil marker) was performed in the pulmonary sections. All data are shown as mean ± SEM. Student’s t-test was performed. Statistical significance is indicated by *p < 0.05, **p < 0.01, and ***p < 0.001.

To further determine the lung inflammatory responses, we examined the secreted pro-inflammatory cytokines and interstitial infiltration of inflammatory cells. At 24 hpi, amounts of inflammatory cytokines (TNF-α, IL-6, IL-1β) and chemokines (KC, CCL2) were higher in the BALF of STING^-/-^ mice than in those of WT mice (**Figures 2A–E**). The inflammatory cytokine IL-6 and chemokines (KC, CCL2) were also higher in lung tissues of STING^-/-^ mice than in those of WT mice (**Figures 2H, J, K**), although IL-1β and TNF-α secretion were comparable between both genotypes (**Figures 2G, I**). The expression levels of CXCL10 in the BALF were similar between the control and infected group (**Figure 2F**), but less induction of IFN-β and CXCL10 by *S. aureus* infection in lung tissues of STING^-/-^ mice in comparison to WT mice (**Figures 2L, M**). In addition, neutrophil accumulation was also elevated in STING^-/-^ mice following *S.aureus* challenge (**Figures 2N, O**). Conclusively, these results suggested that STING deficiency leads to excessive inflammation associated with impaired bacterial clearance during *S.aureus*-induced pneumonia.

### STING Deficiency Also Has a Defect in Bacterial Clearance, but Did Not Exacerbate Pulmonary Inflammation During the Early Stage of Infection

**Figure 3:**
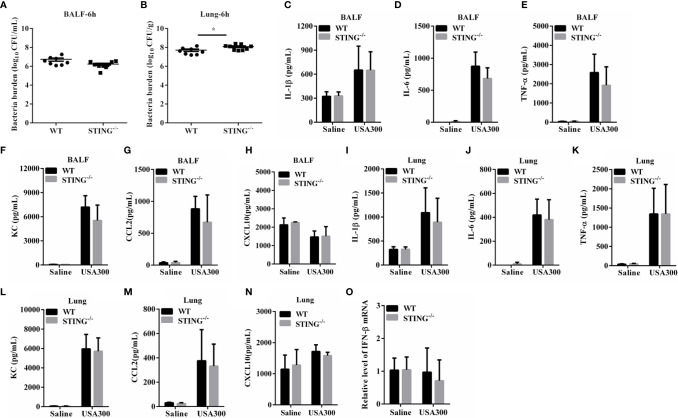
STING promotes early lung bacterial clearance without excessive inflammation. WT and STING^-/-^ mice (N = 8-9/group) were infected intranasally with *S.aureus* (1 × 10^8^ CFU/mouse) and then were euthanized at 6 hpi to quantitate the bacterial burden in **(A)** BALF and **(B)** lung. BALF and lung homogenates were detected for concentrations of indicated cytokines and chemokines by ELISA. **(C, I)** IL-1β, **(D, J)** IL-6, **(E, K)** TNF-α, **(F, L)** KC, **(G, M)** CCL2, and **(H, N)** CXCL10. **(O)** Lung tissue mRNA was examined for IFN-β by qRT-PCR. All data are shown as mean ± SEM. Student’s t-test was performed. Statistical significance is indicated by *p < 0.05, **p < 0.01, and ***p < 0.001.

The increased pulmonary vascular leakage in STING^-/-^ mice on 24 hpi may be the result of exacerbated inflammation or increased bacteria localization. To further investigate the mechanisms for STING mediates protection, we analyzed the initial interplay between bacteria and the host at 6 h after intranasal infection with 1 × 10^8^ CFUs *S.aureus*. Although bacteria loads in the BALF of both genotypes were comparable at 6 hpi (**Figure 3A**), an increased bacterial burden was detected in lung tissues of STING^-/-^ mice compared to WT mice (**Figure 3B**). Excessive inflammation may contribute to the bacteria burden. To assess whether the high bacterial colonization was due to increased inflammatory response in STING^-/-^ mice, cytokine production was examined. The expression levels of inflammatory cytokines (TNF-α, IL-6, IL-1β, IFN-β) and chemokines (KC, CCL2, CXCL10) in BALF and lungs at 6 hpi were comparable between STING^-/-^ and WT mice (**Figures 3C–O**). These results indicate that STING promotes early lung bacterial clearance prior to suppressing excessive inflammation.

### STING-Deficient Mice Present Increased Macrophages Death and Necroptosis Activation Following *S.aureus* Pneumonia

**Figure 4:**
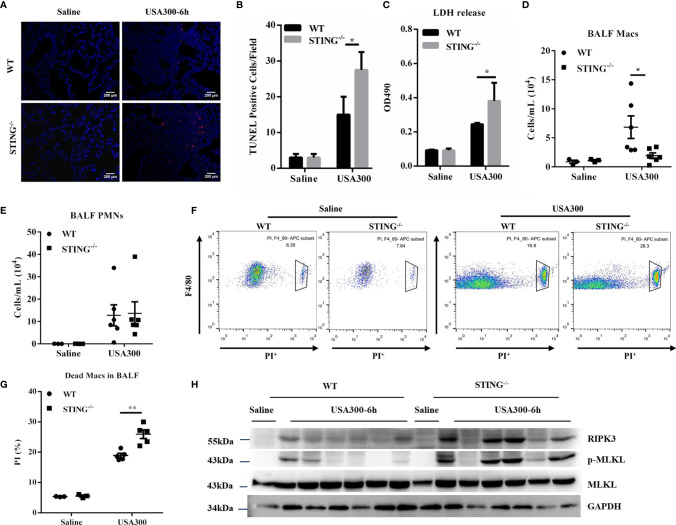
STING^-/-^ mice display increased necroptosis during early *S.aureus* induced pneumonia. WT and STING^-/-^ mice were infected intranasally with *S.aureus* (1 × 10^8^ CFU/mouse) and then were euthanized at 6 hpi. **(A, B)** TUNEL staining of dead cells in the lung tissues. **(C)** LDH released in BALF. **(D, E)** The numbers of neutrophils and macrophages in the BALF were enumerated. **(F, G)** Propidium iodide positive (PI^+^) macrophages in the BALF were analyzed by flow cytometry. **(H)** The lung tissue lysate was analyzed for p-MLKL, MLKL, and RIPK3 by western blotting. GAPDH was used as a loading control. Student’s t-test was performed. Statistical significance is indicated by *p < 0.05 and **p < 0.01.

To understand the mechanism whereby STING restricts early lung bacterial colonization, we sought to investigate whether STING deficiency promoted excessive cell death following *S.aureus* infection. TUNEL staining of histological lung sections showed that STING^-/-^ mice had greatly increased cell death than WT mice (**Figures 4A, B**). Consistent with the increased dead cells in lung sections, STING^-/-^ mice had more LDH release in BALF (**Figure 4C**). We further quantified the number of immune cells in BALF. Compared with WT mice, STING^-/-^ mice had significantly decreased numbers of macrophages in BALF, but equivalent numbers of neutrophils in BALF (**Figures 4D, E**). To better assess macrophage viability, we used flow cytometry to quantify the number of dead macrophages in mouse BALF (F4/80^+^, PI^+^) at 6 hpi. STING^-/-^ mice had more PI^+^ macrophages compared to WT controls (**Figures 4F, G**). Thus, these data suggested that STING is involved in regulating macrophage cell death during pulmonary *S.aureus* infection. As previous research has shown that *S.aureus* induces lung pathology by a distinct cell death mechanism known as necroptosis (29), it is reasonable to speculate whether there is a difference in necroptosis activation that can explain the higher susceptibility of STING^-/-^ mice to *S.aureus* invasion. Given that receptor-interacting-serine-threonine kinase-3 (RIPK3) and mixed lineage kinase-domain like protein (MLKL) are the core initiators and executors of necroptosis (30, 31), RIPK3 and MLKL expression levels were examined to determine necroptosis activation. Our results revealed that RIPK3 and phosphorylation of MLKL markedly increased in the lungs of infected STING^-/-^ mice compared to WT mice at 6 hpi (**Figure 4H**), indicating that STING suppresses macrophage necroptosis activation during pulmonary *S.aureus* infection.

### STING-Deficient Macrophages Display Increased Necroptosis Following *S.aureus* Challenge

**Figure 5:**
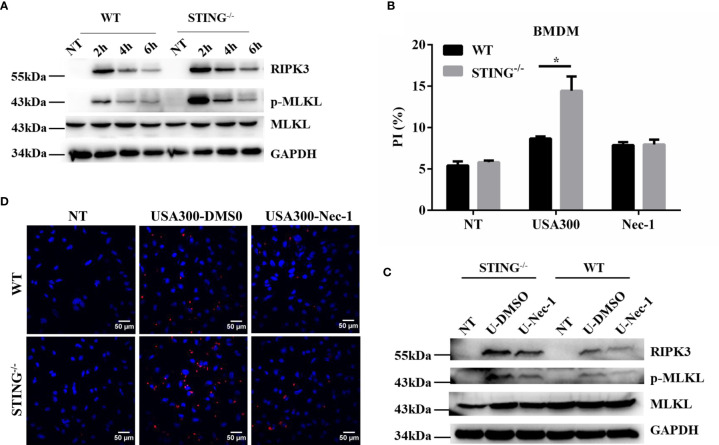
STING reduces activation of necroptosis in macrophages following *S.aureus* challenge. **(A, C)** WT and STING^-/-^ BMDMs were untreated or exposed to *S.aureus* at MOI=50 for the indicated time. BMDMs from WT and STING^-/-^ mice were pre-treated Nec-1 (50 μM) or an equivalent amount of DMSO for 1 h prior to infection with *S.aureus*. The cellular lysate was analyzed for RIPK3, p-MLKL, MLKL, and GAPDH by western blotting. **(B)** Cell death was measured by PI staining in BMDMs infected with *S.aureus* at MOI=50 for 4 h. **(D)** BMDMs from WT and STING^-/-^ mice were infected with *S. aureus* (MOI=50) for 4 hours, p-MLKL activation was observed through fluorescence microscopy. All figures are representative of three independent experiments. Student’s t-test was performed. Statistical significance is indicated by *p < 0.05.

To further characterize the role of STING in *S.aureus* pulmonary infection *in vitro*, the expression of STING in the pulmonary tissues of infected WT mice was investigated by immunohistochemical staining. Brown staining indicates positive reaction. Our results showed that STING was expressed in both uninfected and infected pulmonary tissues of WT mice (**Figure S1**), and primarily located in the inflammatory cells infiltrated to the alveolar spaces. Macrophages are critical in controlling inflammation in the setting of acute *S.aureus* infection and their loss through necroptosis has important pathophysiological consequences in the lung (29). BMDMs isolated from WT and STING^-/-^ mice were infected with *S.aureus* and necroptotic cell death and protein expression were determined. As expected, we found that BMDMs from STING^-/-^ mice exhibited significantly higher levels of RIPK3 and p-MLKL (**Figure 5A**), coupled with increased cell death by PI staining compared with BMDMs from WT mice. To further characterize the mode of cell death, BMDMs from WT and STING^-/-^ mice were pre-treated with necrostatin-1 (Nec-1, necroptosis inhibitor) and then infected with *S.aureus*. Addition of Nec-1 reduced the cell death in STING^-/-^ BMDMs to the level of WT BMDMs while inhibiting MLKL activation (**Figures 5B, C**). Also, we performed immunofluorescence microscopy on BMDMs to quantify p-MLKL expression and found increased p-MLKL- positive cells in BMDMs from STING^-/-^ mice compared to WT KO mice. Pre-treatment of BMDMs with Nec-1 reduced the p-MLKL- positive cells suggesting that the nature of cell death is necroptosis (**Figure 5D**). Meanwhile, the inflammatory cytokines (IL-6, IL-1β, TNF-α) and chemokines (KC, CCL2) secretion were comparable between both groups, the expression levels of CXCL10 and IFN-β were significantly decreased in STING-/-BMDMs (**Figures S2A–F**). These results show that STING has an important role in limiting necroptosis of macrophages, which is consistent with *in vivo* findings.

### MLKL-Deficient Mice Decreases Macrophage Death and Confers Host Resistance to *S.aureus* Infection

**Figure 6:**
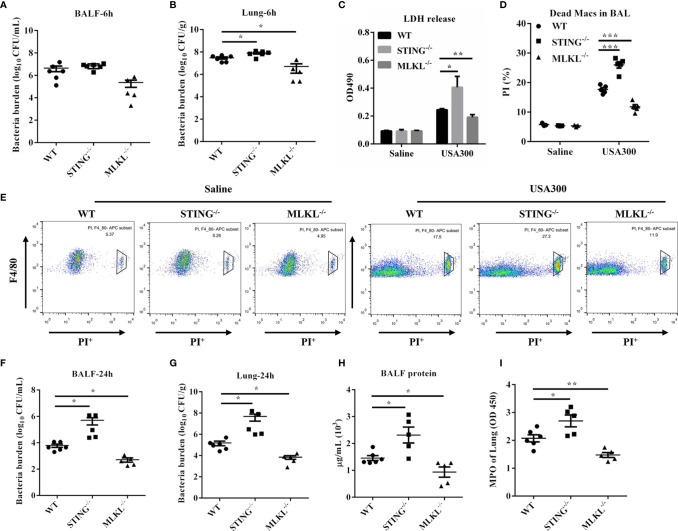
MLKL^-/-^ mice are resistant to pulmonary *S.aureus* infection. WT, STING^-/-^, and MLKL^-/-^ mice (N = 5-6/group) were infected intranasally with a sublethal inoculum of *S.aureus* (1 × 10^8^ CFU/mouse) and then were euthanized at 6 hpi or 24 hpi to quantitate the bacterial burden in **(A, F)** BALF, **(B, G)** lung. **(C)** LDH released in BALF at 6 hpi. **(D, E)** Propidium iodide positive (PI^+^) macrophages in the BALF were analyzed by flow cytometry at 6 hpi. **(H)** Total protein in BALF was measured at 24 hpi. **(I)** The homogenate supernatant of the lung was also used to determine the activity of MPO at 24 hpi. All data are shown as mean ± SEM. One-way ANOVA with Bonferroni’s multiple comparison test was performed. Statistical significance is indicated by *p < 0.05, **p < 0.01, and ***p < 0.001.

MLKL is the critical terminal executioner of necroptosis. To examine whether necroptosis suppression mediates protective effect of STING, we then utilized MLKL^-/-^ mice to evaluate the effect of MLKL on *S.aureus* infection. At 6 hpi, bacteria loads in the BALF of both genotypes were comparable (**Figure 6A**), but a decreased bacterial burden was detected in lung tissues of MLKL^-/-^ mice compared to WT mice (**Figure 6B**). Consistent with the significant difference in the infection phenotype between the WT and MLKL^-/-^ mice, the level of LDH release and the numbers of dead macrophages were also decreased in MLKL^-/-^ mice compared to WT mice (**Figures 6C–E**). At 24 hpi, there was significantly improved *S.aureus* clearance from both BALF and lung in MLKL^-/-^ mice as compared with WT mice (**Figures 6F, G**). Barrier function, as measured by protein content in BALF, was less disrupted (**Figure 6H**), and neutrophil accumulation was also decreased in MLKL^-/-^ mice following *S.aureus* challenge compared with WT mice (**Figure 6I**). These results suggested that MLKL is detrimental to the host followed pulmonary *S.aureus* infection.

### Blocking Necroptosis in STING^-/-^ Mice Augments Host Defense Against *S.aureus* Pneumonia

**Figure 7:**
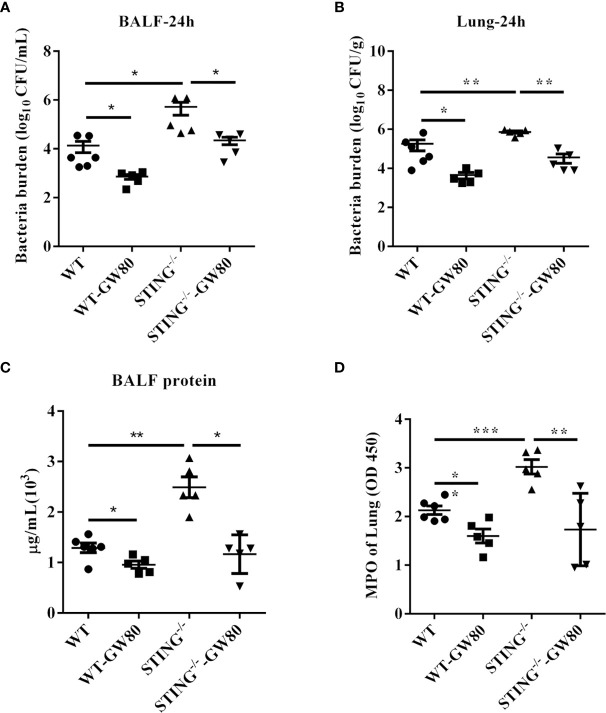
Blocking necroptosis in STING^-/-^ mice improves host defense during *S.aureus* infection. For one group of WT and STING^-/-^ mice, 100 μL of 100 μM GW806742X was injected intraperitoneally 12 h and 1 h prior to infection with *S.aureus* (1 × 10^8^ CFU/mouse). Mice were euthanized at 24 hpi to quantitate the bacterial burden in **(A)** BALF, **(B)** lung. **(C)** Total protein in BALF was measured. **(D)** The homogenate supernatant of the lung was also used to determine the activity of MPO. All data are shown as mean ± SEM. One-way ANOVA with Bonferroni’s multiple comparison test was performed. Statistical significance is indicated by *p < 0.05, **p < 0.01, and ***p < 0.001.

Next, we evaluated whether blocking necroptosis in STING^-/-^ mice rescue impaired host defense against *S.aureus* infection. WT and STING^-/-^ mice were treated intraperitoneally with GW806742X (MLKL inhibitor) or DMSO 12 h and 1 h before infection. GW806742X treatment resulted in reduced bacterial burden in the lungs and BALF of STING^-/-^ mice, which was comparable to that seen in WT mice, indicating that blocking necroptosis benefits bacterial control in STING^-/-^ mice during pulmonary *S.aureus* infection (**Figures 7A, B**). Moreover, blocking necroptosis also decreased the accumulation of neutrophil and the protein content in BALF (**Figures 7C, D**). Collectively, these results showed that STING facilitates pathogen control *via* necroptosis suppression during pulmonary *S.aureus* infection.

## Discussion

*S.aureus* is a common human pathogen associated with respiratory infections ranging from asymptomatic colonization to necrotizing pneumonia. Although antibiotics can restrict bacterial growth and proliferation, these are frequently ineffective in the protection against acute lung injury. Besides, there is no commercially available vaccine for *S.aureus* treatment. Therefore, the need for new preventive and therapeutic strategies has become more urgent. The ultimate outcome of an infection depends on the interaction between the pathogen and the host. Innate immunity is the first line of host defense against pathogenic infections. A detailed understanding of the host innate immune response is critical for the design of potential therapeutic interventions.

As a cytosolic sensor of cyclic dinucleotides, and adaptor molecule for some intracellular DNA sensors, STING plays critical roles in innate immunity. STING activation induces type I interferon responses which mainly participate in effective immune response against viral infections. Concerning bacteria, the outcome of STING activation is controversial, ranging from protective to detrimental effects for the host (23, 32). Thus, rational manipulation of the STING pathway will require careful investigations of host STING-pathogen interactions. Furthermore, the biological implications of STING in *S.aureus* pulmonary infection have not been explored. Using *S.aureus* pneumonia model, we found that STING^-/-^ mice were more susceptible to *S.aureus* infection than WT mice, which was manifested by increased mortality, more bacteria burden in BALF and lungs, severe destruction of lung architecture, increased PMN infiltration, and inflammatory cytokines secretion. These results demonstrated that STING plays an indispensable role in protection against *S.aureus*-induced pneumonia.

Although STING^-/-^ mice at 24 hpi exhibited multiple features including excessive inflammation and increased bacterial burden, it is challenging to delve into the mechanisms underlying the susceptibility to *S.aureus* infection. Infection and inflammation are always intertwined and are mutually the cause or consequence. Increased bacterial load aggravates inflammation, whereas excessive inflammation may exacerbate bacterial colonization. Therefore, to further explore the underlying causes, we analyzed the initial interplay between *S.aureus* and the host at 6 hpi. Intriguingly, we observed a higher *S.aureus* burden in lung tissues of STING^-/-^ mice at the acute stage of infection. Meanwhile, there was no difference in inflammatory cytokines secretion between WT and STING^-/-^ mice. These results suggested that STING contributes to host defense against *S.aureus* lung infection by potentially promoting early bacterial clearance.

Host cell death is an acute immune response against microbial intrusion, which prevents or promotes pathogen replication and survival. By inducing host cell death, bacteria eliminate key immune cells and evade host defenses that can compromise their viability. *S.aureus* toxins such as *agr*, *hla*, *LukAB*, and *psms* are known to deplete alveolar macrophages through activating necroptosis, a mode of cell death, which is a major mechanism of *S.aureus* induced lung damage (29, 33, 34). Main factors that participate in necroptosis-related signal transduction include RIPK1, RIPK3, and its substrate MLKL. Conformational changes induced by phosphorylation result in MLKL N-terminal domain exposure, oligomerization, binding to the membrane, and causing permeabilization and subsequent cell death. We hypothesized that STING may serve as a regulator of necroptosis in the protective response to *S.aureus* infection. The lung sections from STING^-/-^ mice presented more TUNEL-positive cells compared to WT lung sections at 6 hpi. The increased cell death was confirmed by more LDH release in STING^-/-^ BALF. We further quantified the number of immune cells in BALF. Compared with WT mice, STING^-/-^ mice had equivalent numbers of neutrophils in BALF, but a significantly decreased number of macrophages accompanied by increased dead macrophages in BALF. Notably, we found that the infiltration levels of immune cells were different between the early (6 hpi) and late (24 hpi) stages of infection. More dense immune cells (neutrophils) infiltration in STING^-/-^ mice at 24 hpi, which should be attributed to the loss of macrophages through necroptosis during early infection further amplifies the inflammatory response. After all, macrophages comprise the majority of immune cells populating the uninfected lung. Moreover, multiple cell types recruited into the lung in response to infection including neutrophils which are susceptible to necroptosis (35), the ability of the host to replenish most of these cells counteracts necroptotic depletion. Besides, neutrophils are much more avidly phagocytic than macrophages (36), thus their numbers may not influence their total phagocytic clearance. Next, the RIPK3 and phosphorylation of MLKL definitely increased in the lungs of STING^-/-^ mice during early infection compared to the control mice. Due to STING mainly located in the recruited inflammatory cell, BMDMs isolated from WT and STING^-/-^ mice were used to examine the effect of STING on cell death. Consistent with *in vivo* results, increased cell death, and higher levels of RIPK3 and p-MLKL were also presented in infected STING^-/-^ BMDMs compared to infected WT macrophages. The increased cell death in STING^-/-^ BMDMs can be inhibited by the necroptosis inhibitor, Nec-1. Thus, our *in vivo* and *in vitro* findings show that STING inhibits necroptosis activation during *S.aureus* infection. Used MLKL^-/-^ mice, we further show that MLKL^-/-^ mice exhibited decreased macrophage death and enhanced host defense to *S.aureus* infection. This corresponds to findings from other studies, which demonstrate that Rip3^-/-^ mice exhibit significantly improved *S.aureus* clearance and retain an alveolar macrophage population (29). More importantly, blocking necroptosis by MLKL inhibitor rescued host defense defect against *S.aureus* pneumonia in STING^-/-^ mice. Taken together, STING promotes pathogen control *via* necroptosis suppression during pulmonary *S.aureus* infection.

Necroptosis can be initiated by TNF or by TLR3/TLR4 ligands, DNA damaging agents, and T-cell receptor ligation (37). Sarhan et al. first reported that pre-established IFN status is critical for the early initiation of necroptosis in macrophages, during which needed the participation of cGAS/STING (38). Subsequently, Brault et al. corroborated that cGAS-STING sensing intracellular DNA leads to necroptosis depending on type I IFN (39). Here, our study provided a novel discovery of STING in the context of *S.aureus* pneumonia, which inhibits macrophage necroptosis to strengthen bacterial control and host defense.

In conclusion, the present study reveals the beneficial role of STING during *S.aureus* pneumonia. STING promotes host survival and augments bacteria eradication before developing excessive inflammation. Moreover, STING inhibits necroptosis activation to augment host defense. Considering that *S.aureus* toxins-induced necroptosis is the major cause of lung damage. It would be interesting to see if STING regulates toxins-triggered cellular damage. This may contribute to prompt further questions of host-pathogen interaction during *S.aureus* infection.

## Data Availability Statement

The original contributions presented in the study are included in the article/**Supplementary Material**. Further inquiries can be directed to the corresponding author.

## Ethics Statement

All animal studies were conducted according to experimental practices and standards approved by the Animal Welfare and Research Ethics Committee at Jilin University (No. 20150601).

## Author Contributions

WC, Y-JY, and Z-ZL designed experiments. Z-ZL, Y-JY, C-KZ, S-QY, KM, and YG performed the experiments and analyzed the data. Z-ZL, WC, and Y-JY wrote the manuscript. All authors contributed to the article and approved the submitted version.

## Funding

This work was supported by National Natural Science Foundation of China (No. 31872457, No. 31972682).

## Conflict of Interest

The authors declare that the research was conducted in the absence of any commercial or financial relationships that could be construed as a potential conflict of interest.

## Supplementary Material

Supplementary Figure 1 STING expression in the lung tissues. WT and STING^-/-^ mice were infected intranasally with *S.aureus* (1 × 10^8^ CFU/mouse) and then were euthanized at 24 hpi. Representative immunohistochemical staining of STING was performed in the pulmonary sections.

Supplementary Figure 2 Analyzing the role of STING in producing cytokines/chemokines after *S. aureus* infection. WT and STING^-/-^ BMDMs were treated with *S.aureus* at a MOI=50 for 6 h. **(A–F)** Culture supernatants from BMDMs were analyzed for IL-1β, IL-6, TNF-α, KC, CCL2, and CXCL10 by ELISA. **(G)** IFN-β mRNA levels were measured at 6 hpi by qRT-PCR. All data are shown as mean ± SEM. Student’s t-test was performed. Statistical significance is indicated by * p < 0.05, ** p < 0.01.

## Abbreviations

*S.aureus*, *Staphylococcus aureus*; STING, stimulator of interferon genes; PRRs, pattern recognition receptors; IFI204, interferon-inducible protein 204; cGAS, cyclic GMP-AMP synthase; ZBP1, Z-DNA binding protein 1; DDX41, DEAD-box helicase 41; RIPK3, receptor-interacting-serine-threonine kinase-3; MLKL, mixed lineage kinase-domain like protein; TBK1, TANK binding kinase 1; IRF3, interferon regulatory factor 3; MOI, multiplicity of infection; BMDM, bone marrow-derived macrophage; CFU, colony-forming unit; H&E, hematoxylin and eosin.
