# Supplementary figures and images for: STING Contributes to Host Defense Against Staphylococcus aureus Pneumonia Through Suppressing Necroptosis
Source: Front Immunol. 2021 May 31;12:636861. doi: 10.3389/fimmu.2021.636861 (PMC8202078; doi:10.3389/fimmu.2021.636861)

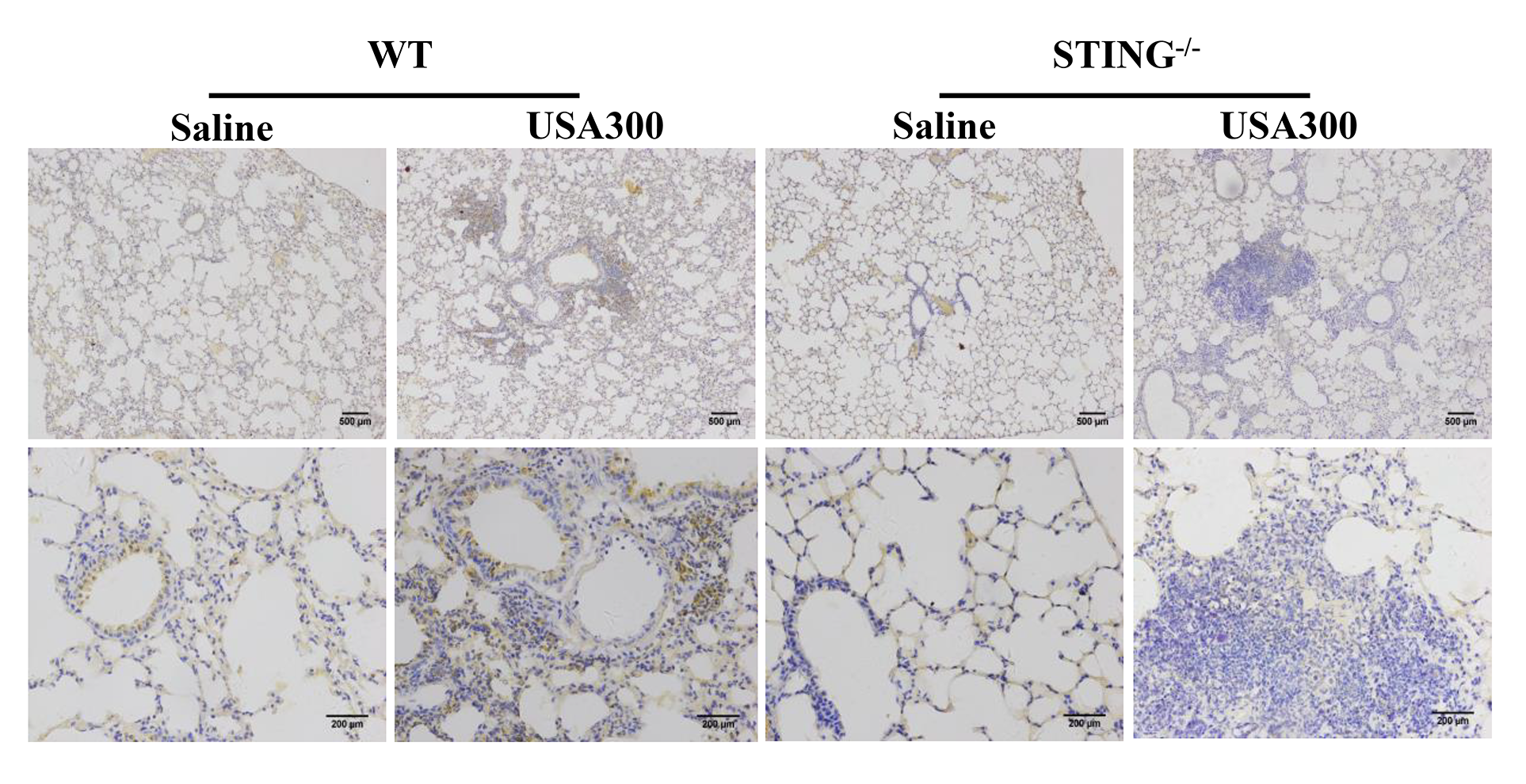

Supplement: Supplementary Figure 1 — STING expression in the lung tissues. WT and STING-/- mice were infected intranasally with S.aureus (1 × 10^8^ CFU/mouse) and then were euthanized at 24 hpi. Representative immunohistochemical staining of STING was performed in the pulmonary sections. [file Image_1.tif]

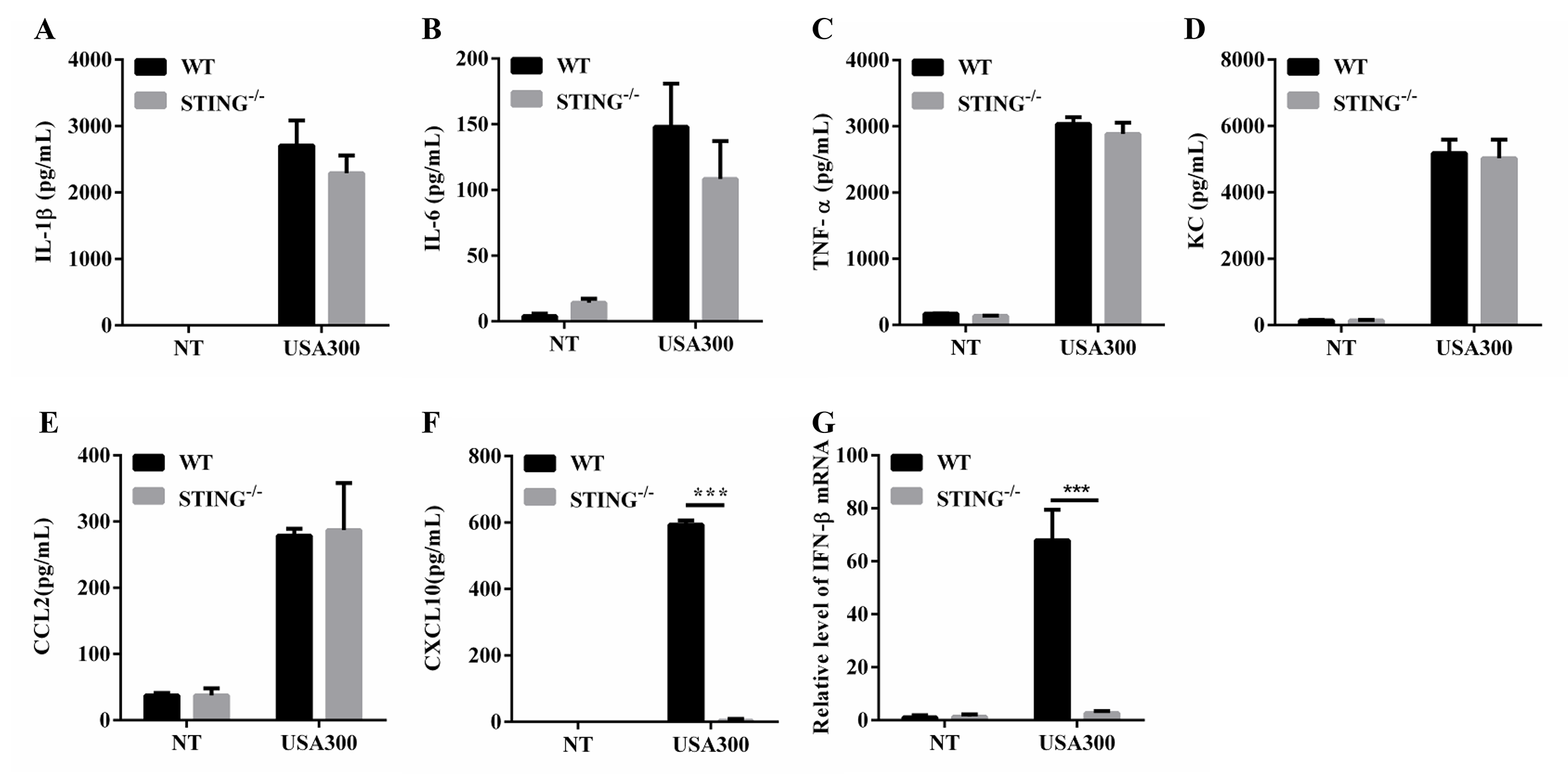

Supplement: Supplementary Figure 2 — Analyzing the role of STING in producing cytokines/chemokines after S. aureus infection. WT and STING-/- BMDMs were treated with S.aureus at a MOI=50 for 6 h. (A–F) Culture supernatants from BMDMs were analyzed for IL-1β, IL-6, TNF-α, KC, CCL2, and CXCL10 by ELISA. (G) IFN-β mRNA levels were measured at 6 hpi by qRT-PCR. All data are shown as mean ± SEM. Student’s t-test was performed. Statistical significance is indicated by * p < 0.05, ** p < 0.01. [file Image_2.tif]
